# Validation of diagnostic nomograms based on CE–MS urinary biomarkers to detect clinically significant prostate cancer

**DOI:** 10.1007/s00345-022-04077-1

**Published:** 2022-07-16

**Authors:** Maria Frantzi, Isabel Heidegger, Marie C. Roesch, Enrique Gomez-Gomez, Eberhard Steiner, Antonia Vlahou, William Mullen, Ipek Guler, Axel S. Merseburger, Harald Mischak, Zoran Culig

**Affiliations:** 1 Department of Biomarker Research, Mosaiques Diagnostics, Hannover, Germany; 2 Experimental Urology Department of Urology, Medical University of Innsbruck, Anichstrasse 35, 6020 Innsbruck, Austria; 3 Department of Urology, University Hospital Schleswig-Holstein, Campus Lübeck, Lübeck, Germany; 4 Urology Department, Reina Sofía University Hospital, Maimonides Institute of Biomedical Research of Cordoba (IMIBIC), University of Cordoba (UCO), Cordoba, Spain; 5 Systems Biology Center, Biomedical Research Foundation, Academy of Athens, Athens, Greece; 6 Institute of Cardiovascular and Medical Science, University of Glasgow, Glasgow, UK; 7 Leuven Biostatistics and Statistical Bioinformatics Centre (L-BioStat), Katholiek Universiteit (KU) Leuven, University of Leuven, Leuven, Belgium

**Keywords:** Prostate cancer aggressivity, Non-invasive urine biomarker, Capillary electrophoresis, Urinary peptide marker

## Abstract

**Purpose:**

Prostate cancer (PCa) is one of the most common cancers and one of the leading causes of death worldwide. Thus, one major issue in PCa research is to accurately distinguish between indolent and clinically significant (csPCa) to reduce overdiagnosis and overtreatment. In this study, we aim to validate the usefulness of diagnostic nomograms (DN) to detect csPCa, based on previously published urinary biomarkers.

**Methods:**

Capillary electrophoresis/mass spectrometry was employed to validate a previously published biomarker model based on 19 urinary peptides specific for csPCa. Added value of the 19-biomarker (BM) model was assessed in diagnostic nomograms including prostate-specific antigen (PSA), PSA density and the risk calculator from the European Randomized Study of Screening. For this purpose, urine samples from 147 PCa patients were collected prior to prostate biopsy and before performing digital rectal examination (DRE). The 19-BM score was estimated via a support vector machine-based software based on the pre-defined cutoff criterion of − 0.07. DNs were subsequently developed to assess added value of integrative diagnostics.

**Results:**

Independent validation of the 19-BM resulted in an 87% sensitivity and 65% specificity, with an AUC of 0.81, outperforming PSA (AUC _PSA_: 0.64), PSA density (AUC _PSAD_: 0.64) and ERSPC-3/4 risk calculator (0.67). Integration of 19-BM with the rest clinical variables into distinct DN, resulted in improved (AUC range: 0.82–0.88) but not significantly better performances over 19-BM alone.

**Conclusion:**

19-BM alone or upon integration with clinical variables into DN, might be useful for detecting csPCa by decreasing the number of biopsies.

**Supplementary Information:**

The online version contains supplementary material available at 10.1007/s00345-022-04077-1.

## Introduction

Prostate cancer (PCa) ranks as the second most frequent and the fifth leading cause of cancer death among men [1]. In 2020, almost 1.4 million new cases were diagnosed worldwide, and 375 000 deaths were reported due to PCa. Although this malignancy is diagnosed in 15–20% of men, the lifetime risk of death is significantly lower (3%) [2]. For patients presenting with slow growing PCa, defined according to the European Association of Urology (EAU) guidelines as clinically insignificant cancer (insPCa: Gleason score, GS < 7 and PSA < 10 ng/ml and max 2 cores) [3], immediate treatment is not recommended, rather management by active surveillance (AS) including re-biopsies in certain time frames [3].

Screening for PCa is currently based on serum prostate-specific antigen (PSA) testing and digital rectal examination (DRE). However, multiple factors not related to prostate malignancy may affect the level of serum PSA [4]; thus, less than half of patients with elevated PSA are consequently confirmed with PCa [5]. Therefore, intensive PSA screening has led to the identification of numerous insPCa often associated with overtreatment. Definitive diagnosis of PCa is based on the histopathological confirmation of PCa in biopsy cores, following a positive result of DRE and/or high PSA levels [3]. Until recently, the procedure was guided only by transrectal ultrasound (TRUS) [6]. In an effort to improve the accuracy for PCa detection, multiparametric magnetic resonance imaging (mpMRI) has been recently adopted, resulting in good sensitivity for detecting GS ≥ 3 + 4 (sensitivity of 91%, specificity of 37%) [7]. Nevertheless, mpMRI is less sensitive for GS < 3 + 4 (sensitivity of 70%, specificity of 27%) [7]. Moreover, while mpMRI is beneficial particularly for guiding repeated biopsy [8], inter-reader variability among radiologists as well as the limited capacity to perform a high number of MRI-guided procedures remain significant challenges [9].

High-throughput *-omics* technologies allow for simultaneous acquisition of thousands of features and a better definition of molecular pathophysiology in cancer [10]. In the context of PCa, various candidate biomarkers have been described [11] with single biomarkers frequently lacking diagnostic accuracy for routine clinical application [12]. In this context, high-resolution urinary proteomics profiles from > 800 patients had been previously acquired by capillary electrophoresis coupled to mass spectrometry (CE–MS). Subsequently, proteomics patterns that were developed using machine learning algorithms in a form of a 19-biomarker model (19-BM) were developed to discriminate csPCa (GS ≥ 7) from slow-progressing PCa in patients with low PSA levels (< 15 ng/ml) [13]. Based on the previously published data [13], the 19-BM resulted in a 90% sensitivity and 59% specificity with an AUC of 0.81, outperforming PSA (AUC_PSA_: 0.58) and the ERSPC-3/4 risk calculator (AUC_ERSPC_: 0.69). Moreover, based on a first investigation, integration of the CE–MS biomarkers with other variables such as PSA and age showed an increased performance (AUC: 0.83), demonstrating a level of complementarity between the tests. Considering this evidence, in this study, the aim was to validate the previously established CE–MS-based 19-BM, and additionally investigate whether integrative models can lead to improved non-invasive and more accurate discrimination between insPCa and csPCa.

## Materials and methods

This study was performed according to the REMARK Reporting Recommendations [14] and the recommendations for biomarker identification and reporting in clinical proteomics [15], including 148 patients who underwent a transrectal ultrasound (TRUS)-guided prostate biopsy based on suspicion for PCa [16] at the Department of Urology of Medical University of Innsbruck, between 2005 and 2012. Sample collection and processing were approved by the local Ethics Committee at Innsbruck Medical University (reference number: 11438/2017) and informed consent was obtained from all participants. Of the 148 patients for whom biopsy results confirmed presence of adenocarcinoma of prostate, one patient was excluded as the PSA measurement was missing. A schematic representation of the study design is presented in Supplementary Figure. D'Amico classification utilizing Gleason Score (GS), PSA criteria [3, 17] and T-stage were applied to classify the PCa patients into risk groups (low, intermediate and high).

At the time of patient enrollment, mpMRI-guided biopsy was not yet implemented in the clinical practice, therefore, all patients underwent TRUS‐guided biopsy and 15-biopsy cores were obtained. Full clinical and laboratory data were collected and are presented in the Supplementary Table S1*.* The European Randomised Study of Screening for Prostate Cancer (ERSPC) estimates for risk stratification were calculated as previously described [18], considering serum PSA levels, the DRE result and information about the previous biopsies (https://www.prostatecancer-riskcalculator.com). The patient cohort characteristics are summarized in *Table **1**.*

**Table 1:** Clinical and biochemical variables for the 147 patients with confirmed PCa

Baseline characteristics	Patients with confirmed PCa (*n* = 147)
Median age (95% CI; yr)	**66.0** (64.2–67.0)
Age range (yr)	**40–84**
PSA median (95% CI; ng/ml)	**5.2** (4.5–5.9)
Digital rectal examination (normal/suspicious/NA)	**90/20/37**
Previous biopsies (Y/N)	**43/104**
Median urinary creatinine (95% CI; mmol/L)	**8.8** (7.6–10.3)
Median total protein (95% CI; mmol/L)	**0.03** (0.03–0.05)
Disease pathology	
GS 6	**99**
GS 3 + 4/4 + 3	**31/4**
GS 8	**8**
GS 9	**5**
D’amico risk stratification	
Low risk	**80**
Intermediate risk	**44**
High risk	**17**
Significant PCa (GS ≥ 3 + 4)	**48** (32.7%)
Insignificant PCa (GS: 6)	**99** (67.3%)

*95% CI* Confidence interval, *GS* Gleason score, *NA* Data not available, *N* Not received, *PCa* Prostate cancer, *Y* Received

### Urine collection and mass spectrometry analysis

All urine samples were collected prior to prostate biopsy according to clinical guidelines and without performing DRE before biopsy. Voided urine samples were collected in sterile containers and immediately stored at – 20 ℃ until further processing. Sample preparation and mass spectrometry analysis was performed as described in detail in *Supplementary Text.*

### Statistical evaluation of model predictivity

The proportion, mean, standard deviation, median and interquartile range (25–75th percentiles) estimates were calculated to describe the distribution of the different variables in the patient cohort (summarized in Table 1). The biomarker model’s scores were calculated via the support vector machine (SVM)-based software, namely MosaCluster (version1.7.0), as previously described [19]. A detailed description is given in Supplementary Text and the list of scoring data is presented in Supplementary Table S2.

## Results

### Validation of 19-BM based on CE–MS urinary peptide biomarkers

**Fig. 1:**
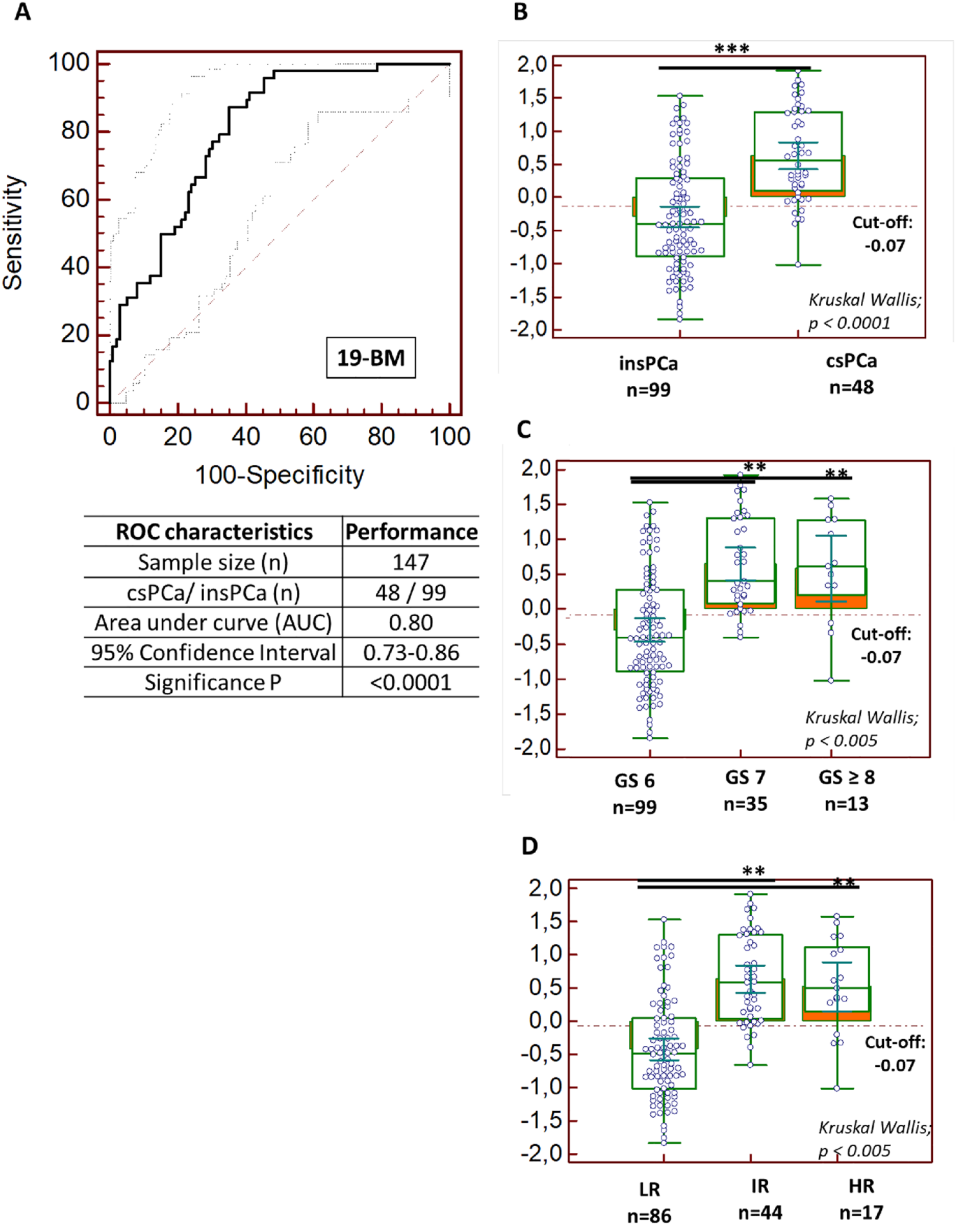
**A** Receiver-operating characteristics (ROC) analysis displaying the performance of the 19-biomarker model for discriminating csPCa from nsPCa; **B** classification scores, presented in Box-and-Whisker plots grouped according to the csPCa (*n* = 48) and nsPCa (*n* = 99), **C** classification scores displaying the level of discrimination across the different Gleason score, and **D** risk groups based on D’Amico classification. A post hoc rank-test was performed using Kruskal–Wallis test

**Fig. 2:**
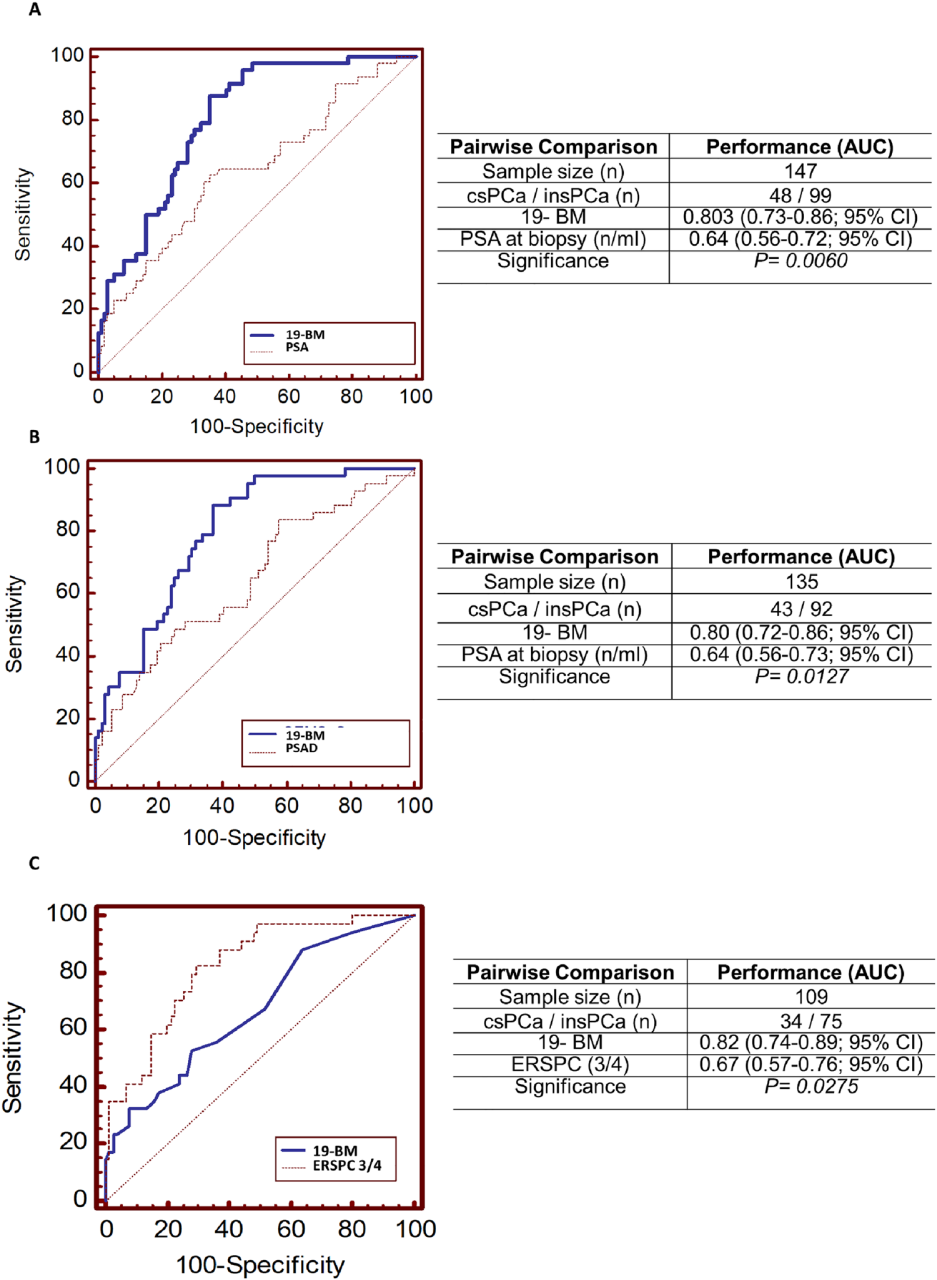
Comparative analysis depicted by receiver-operating characteristics (ROC) curves of the 19-biomarker model (19-BM) with **A** serum PSA measurements, **B** PSA density (PSAd) and **C** for the 19-BM and the ERSPC-3/4 (considering a subgroup of 109 PCa patients for whom available clinical data enabled ERSPC estimation)

First, the performance of the previously published 19-BM [13] was validated in line with the recommendations for biomarker identification and reporting in clinical proteomics [15]. Considering 99 patients with insPCa and 48 patients with csPCa, the AUC for the 19-BM (AUC_19-BM_) was estimated at 0.803 with the 95% CI ranging from 0.73 to 0.86 (*p* < *0.0001*). At the validated cutoff level of -0.07 [13], the sensitivity was estimated at 87.5% and the specificity at 64.6% (Fig. 1A). The 19-BM correctly classified 42 out of the 48 csPCa (GS ≥ 3 + 4), whereas 35 out of the 99 insPCa (GS: 6) were misclassified as csPCa cases. Considering a prevalence rate of 32.7 for csPCa, as estimated based on this patient cohort, the negative predictive value (NPV) for detecting csPCa was computed at 91.8% (71.9–99.1%; 95% CI) while the positive predictive value (PPV) was 55.0% (34–75.8%; 95% CI). Moreover, the 19-BM significantly discriminated between significant and insignificant PCa (*p* < *0.0001*, Kruskal–Wallis H test, Fig. 2B), separated patients with GS: 6 from both those with GS: 7 and GS ≥ 8 (*p* < *0.005*, Kruskal–Wallis H test; Fig. 1C) but also according to the risk group (*p* < *0.005*, Kruskal–Wallis H test; Fig. 1D). In addition, the classification based on the urinary 19-BM was not affected by biochemical variables such as urinary creatinine (Spearman’s rank correlation coefficient: 0.104; − 0.06 to 0.27; 95% CI; *p* = 0.2278) and total urinary protein (Spearman’s rank correlation coefficient: − 0.058; − 0.22 to 0.11, 95% CI; *p* = 0.5060).

### Comparative assessment of 19-BM with PSA and PSA density

Considering serum PSA levels that were measured at the time point of biopsy, a direct comparison of PSA and PSA density (PSAd) with the CE–MS-based 19-BM was performed. Multivariate analysis showed that the 19-BM outperformed PSA in discriminating csPCa (AUC_PSA:_ 0.64; 0.55–0.72; 95% CI; *p* = 0.006*; *Fig. 2A). The sensitivity of the PSA was estimated at 66.7% (51.6–79.6%; 95% CI; PSA > 4 ng/ml), while the specificity at 44.4% (34.5–79.6; 95% CI; PSA > 4 ng/ml). Sixteen patients with csPCa were detected by the 19-BM but were missed by serum PSA. Out of 16 patients, 13 were bearing GS: 3 + 4 PCa tumors, but were also three patients with GS ≥ 4 + 3. In addition, both the NPV (73.3; 48.4–90.6; 95% CI) and the PPV (36.8; 19.5–57.0; 95% CI) estimates were lower than the estimates based on the 19-BM. Considering 135 PCa patients, for whom data on prostate volume were available, a direct comparison of the 19-BM with PSAd was also possible. As in the case of serum PSA, the 19-BM outperformed PSAd in discriminating csPCa (AUC_PSAd:_ 0.64; 0.56–0.73; 95% CI; *p* = 0.0127; Fig. 2B). The sensitivity of the PSAd was estimated at 83.7% (69.3–93.2%; 95% CI), while the specificity at 42.4% (32.1–53.1; 95% CI). Six patients with csPCa were detected by the 19-BM but were missed when considering PSAd. Out of seven patients, five were bearing GS: 3 + 4 PCa, but were also two patients with GS ≥ 4 + 3. Similarly, to the PSA comparison, both the NPV (84.4; 55.6–97.8; 95% CI) and the PPV (41.3; 23.2–61.4; 95% CI) estimates for PSAd were lower than the estimates based on the 19-BM.

### Comparison of 19-BM with the ERSPC clinical risk calculator

In order to investigate if the 19-BM can improve on the current state-of-the-art clinical prognosticators, the SVM-based scores from 19-BM were further compared with the estimates of ERSPC risk calculator for detecting high risk PCa (ERSPC-3/4), as presented in Fig. 2C. Based on the available clinical data for the PSA levels, the DRE result and accounting also for the previous biopsies, ERSPC-3/4 estimates for 109 PCa patients were available for this comparison. In addition, based on this analysis, the performance of 19-BM (_AUC19-BM_ = 0.82; 0.74–0.89) was significantly higher than the one of ERSPC-3/4 risk calculator (AUC_ERSPC3/4_ = 0.67; 0.57–0.76; *p* = 0.0275). At the optimal cutoff level, sensitivity of the ERSPC-3/4 was estimated at 52.9% (35.1–70.2; 95% CI) and specificity at 72.0% (60.4–81.8; 95% CI). Fifteen of the 34 patients with confirmed csPCa, including 3 GS ≥ 8 tumors were misclassified by the ERSPC-3/4. Interestingly, 14 of the 15 were detected by the 19-BM. Considering the predictive values, both the NPV (75.9; 52.5–91.6; 95% CI) and the PPV (47.9; 19.6–77.3; 95% CI) estimates were slightly but not significantly lower than the estimates based on the 19-BM.

### Integrative diagnostics: assessment of multimodal models based on 19-BM

**Fig. 3:**
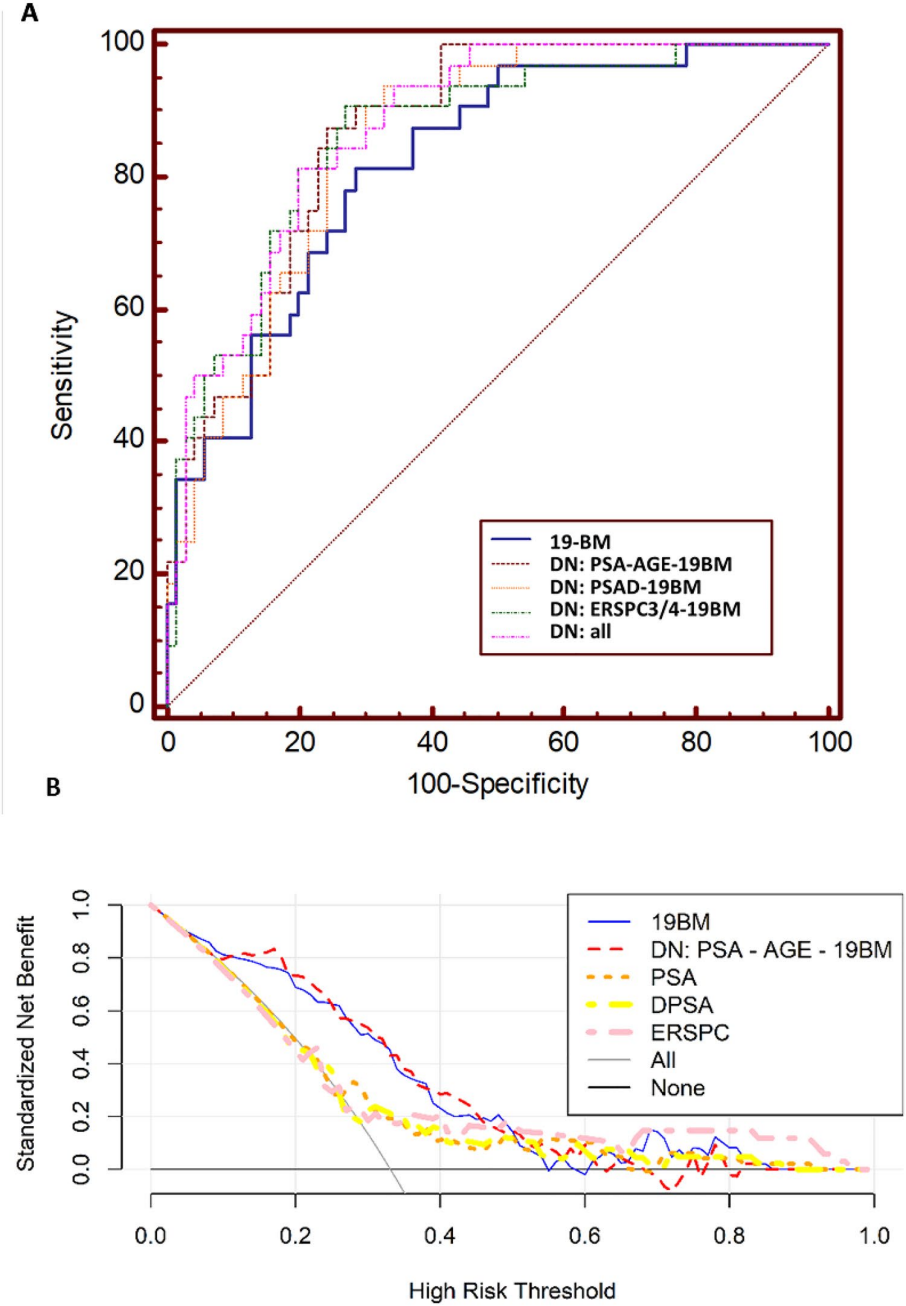
**A** Receiver-operating characteristics (ROC) analysis displaying the performance of DN nomograms based on 19-BM combined with the state-of-the-art risk variables (PSA, PSAd, ERSPC-3/4 and age). **B** Results of the decision curve analysis, comparing the net benefit for the prediction of csPCa on biopsy using the 19BM (blue line), and DN (PSA-AGE-19BM; red line), PSA (orange line), DPSA (yellow line) and ERSPC high risk 3/4 (pink line) as a function of the risk threshold, compared to those benefits of the strategies of treating all patients (grey line) and treating none (black line)

As before [13], in this study, integration of PSA and age with the 19-BM (namely DN_PSA-AGE-CE_) resulted in an improved AUC value of 0.83 (0.76–0.89; 95% CI), although not statistically significant (*p* = 0.1308) compared to the 19-BM (AUC: 0.80; 0.73–0.87; Fig. 3*A*). Yet, the integrative nomogram outperformed PSA (AUC: 0.64; 0.5–0.72; 95% CI; *p* = 0.0001; Table 2). In addition, a second DN was developed by integrating PSAd and 19-BM (DN_PSAd-19BM_), performing once again slightly (AUC_DN:PSAd-CE_: 0.82; 0.74–0.88; 95% CI) but not significantly better than 19-BM alone (*p* = 0.2421; Fig. 3A; Table 2). In comparison to PSAd alone, DN_PSAD-19BM_ demonstrated significantly better performance based on the ROC pairwise comparison (*p* = 0.001*).* Similarly, a third DN was evaluated by integrating ERSPC-3/4 with 19-BM (DN_ERSPC3/4-19BM_). Although the integrative performance for DN_ERSPC3/4-19BM_ was even higher reaching an AUC of 0.86 (0.78–0.92; 95% CI), the difference was again not statistically significant when compared to 19-BM alone (*p* = 0.076; Fig. 3A; Table 2). Lastly, all the above significant clinical variables were integrated together into a DN including all relevant risk estimates such as the 19-BM, PSA, PSAd, age and ERSPC-3/4. The performance for this DN including all significant variables was further improved reaching an AUC of 0.88 (0.80–0.93; 95% CI), significantly outperforming PSA (*p* = 0.002), PSAd (*p* < 0.001), ERSPC-3/4 (*p* = 0.0007*)* alone but not the 19-BM (*p* = 0.06) alone (Fig. 3A). Considering the optimal cutoff for the DN (> 0.1766; Youden index), the sensitivity was estimated at 93.8% (79.2–99.2; 95% CI) and the specificity at 65.7% (53.4–76.7; 95% CI). The integrative nomogram correctly classified 30 out of the 32 significant PCa (GS ≥ 3 + 4) and misclassified two patients with GS: 7. In addition, the NPV for detecting csPCa was computed at 95.6% (71.3–99.9%; 95% CI), whereas the PPV at 57% (32–79.6%; 95% CI). To assess the clinical benefit of the integrative model, a decision curve analysis was performed. Based on the net plotting against the threshold probabilities for the comparisons between the DN and the 19-BM, PSA, PSAd and ERSPC estimates, there is a clear benefit of the integrative model particularly in the lower range of the risk thresholds (Fig. 3B).

**Table 2:** Summary of pairwise statistical comparisons for the developed integrative diagnostic nomograms (DN)

	AUC	95% CI	*P* value
DN: PSA-age-19BM	0.83	0.76–0.89	
19BM	0.81	0.73–0.87	*0.1308*
PSA	0.64	0.56–0.72	***0.0001***
DN: PSAd-19BM	0.82	0.74–0.88	
19BM	0.80	0.72–0.86	*0.2421*
PSAd	0.64	0.56–0.73	***0.0001***
DN: ERSPC-3/4-19BM	0.86	0.78–0.92	
19BM	0.82	0.74–0.89	*0.076*
ERSPC-3/4	0.67	0.57–0.76	***0.001***
DN: all variables	0.88	0.80–0.93	
19BM	0.82	0.73–0.89	*0.0645*
PSA	0.69	0.60–0.78	***0.0002***
PSAd	0.65	0,56–0.75	** < *0.0001***
ERSPC-3/4	0.69	0.59–0.78	***0.0007***

## Discussion

Accurate and frequent monitoring is required to detect clinically relevant disease in a timely fashion. Routine monitoring is commonly based on either PSA levels in addition to regular mpMRI and invasive re-biopsies. To delay or even avoid unnecessary biopsies, overdiagnosis and overtreatment, biomarkers to guide biopsy are of value. *-omics* based studies have been published reporting on discriminatory features of PCa biopsy outcome to guide prostate biopsy [20, 21] Using CE–MS proteomics, a biomarker model based on 19 urinary peptides (19BM) was established with the aim to accurately detect csPCa and validated in 823 patients suspicious for PCa (reporting an AUC of 0.81, outperforming PSA and ERPSC) [13]. Considering urine collection without prior DRE, and building upon this previous report, in this study, we aimed at validating the 19-BM in 147 patients with confirmed PCa. The 19-BM demonstrated high reproducibility in this external validation study for discriminating significant PCa. The 19-BM exhibited good performance (AUC_19-BM_: 0.80) that was comparable to the previously described estimates (AUC: 0.81). Furthermore, the 19-BM sensitivity (88%) and specificity (65%) were similar to those previously reported (90% and 59%, respectively) [13]. The clinical consequence of this observation can be weighted as tolerable, since patients with positive score based on the CE–MS assessment will further undergo biopsy to rule out the presence of csPCa. In this study, 19-BM again performed better than PSA and successfully classified most of PCa cases (19-BM: 42/48 patients; PSA: 32/48 patients). Moreover, both the NPV of 19-BM and the PPV were also higher than PSA respective estimates. The same observations were reported when comparing to PSAd (AUC: 0.64) and the ERSPC-3/4 calculator (AUC: 0.67).

To investigate if improved performance is reported upon combination of 19-BM with current state-of-the-art risk calculators, several integrative diagnostics strategies were employed to develop DN including different combinations of 19-BM with the significant clinical variables such as PSA, PSAd, age, ERSPC. In all above comparisons, the integrative DNs were demonstrating improved performance, an observation which is in line with previous evidence for a level of complementarity of the diagnostics assays. Yet, in all comparisons, the performance of the multimodal DNs was not significantly better than the 19-BM alone.

Considering the above scientific evidence, the very high NPV (> 90%), as well as the fact that for the 19-BM test, there is no need for performing a DRE before urine collection, the specific clinical impact of such a non-invasive test like 19-BM or a DN based on 19-BM, would primarily be to guide biopsy and eventually reduce the number of invasive biopsies in primary PCa diagnosis PATHWAY?. The required high sensitivity for accurate detection of csPCa was achieved in this study. Upon potential application of such a test and in view of a positive test, urologists are alerted to perform a more thorough investigation, improving the overall accuracy in detection of csPCa. Lower specificity would likely result in more misclassifications of an insPCa form as a csPCa. As a result, a positive result based on 19-BM or a DN based on 19-BM should be complemented by MRI to rule out csPCa.

Considering the literature, several biomarkers have been tested in order to discriminate csPCa, such as 4 K score test, PHI, PCA3, SelectMDx) [22, 23]. The PCA3 urinary assay demonstrated 67% sensitivity and 83% specificity for detecting PCa [24]. In comparison to the PHI in guiding initial and repeated biopsy, the PCA3 assay performed slightly, but not significantly inferiorly in both the initial (AUC_PCA3_: 0.57; AUC_PHI_: 0.69) and the repeated biopsy setting (AUC_PCA3_: 0.63; AUC_PHI_: 0.72) [25]. SelectMDx assay demonstrated an AUC of 0.73 [26], while Mi-Prostate Score resulted in an AUC of 0.76 for detection of PCa [27]. The validation results shown in this study with an AUC > 0.80 and > 0.90 for the integrative nomogram are higher than the range 0.57–0.73 which is shown by other biomarkers and clearly justify implementation of this approach in a future investigative setting. Moreover, for many urinary biomarkers, performing a DRE is crucial as it increases the excretion of fluid from the prostate. Yet, for the 19-BM test, a DRE is not a prerequisite for the analysis.

Yet, the study also presents with certain limitations. First, a direct comparison with the above biomarkers reported in the literature was unfortunately not possible, as paired data were not available. Moreover, this study was performed retrospectively, however, on samples that were prospectively collected. Nevertheless, based on the data presented, implementation in an investigative setting seems to be highly justified. As another limitation, also pMRI data were not available for this patient cohort. 43 out of 104 patients in our study had previous biopsies. However, the samples were collected between 2005 and 2012, at the time when mpMRI was not an established standard in primary or repeated biopsies. To facilitate comparisons and inclusion of multiparametric MRI, validation in a future prospective setting is planned.

Collectively, the data presented in this study could demonstrate the utility of a multimodal approach for improved non-invasive detection of significant PCa. Considering the high NPV, the clinical utility of the presented nomogram could also be potentially investigated in the context of guiding mpMRI.

## Supplementary Information

Below is the link to the electronic supplementary material.

Supplementary file1 Supplementary Table S1: Clinical and demographical data for the 147 patients with confirmed PCa (PDF 545 KB)

Supplementary file2 Supplementary Table S2: List of scoring data for 147 patients with PCa, including the SVM-based and the nomogram scoring data (PDF 462 KB)

Supplementary file3 Supplementary Figure: Schematic representation of the study design for the validation of urine CE-MS based nomograms. (TIF 230 KB)

Supplementary file4 Supplementary Text: Urine sample processing, mass spectrometry analysis and performance evaluation (DOCX 26 KB)

## Data Availability

All data associated with our manuscript including clinical demographical and SVM scoring data, together with details of the software parameters are included in the manuscript. Mass spectrometry raw data can be provided upon request.
