# Supplementary material for: Validation of diagnostic nomograms based on CE–MS urinary biomarkers to detect clinically significant prostate cancer
Source: World J Urol. 2022 Jul 16;40(9):2195–203. doi: 10.1007/s00345-022-04077-1 (PMC9427869; doi:10.1007/s00345-022-04077-1)
Supplement: Supplementary file 1 — Supplementary file1 Supplementary Table S1: Clinical and demographical data for the 147 patients with confirmed PCa (PDF 545 KB) [file 345_2022_4077_MOESM1_ESM.pdf]

CE-MS_Analysis ID	Age	Diagnosis	Sample	Gender	Risk Assessment
189008	66	Prostate Carcinoma	Urine	male	low risk
189022	72	Prostate Carcinoma	Urine	male	interm risk
189023	66	Prostate Carcinoma	Urine	male	low risk
189024	77	Prostate Carcinoma	Urine	male	low risk
189025	73	Prostate Carcinoma	Urine	male	interm risk
189026	61	Prostate Carcinoma	Urine	male	interm risk
189027	78	Prostate Carcinoma	Urine	male	interm risk
189033	59	Prostate Carcinoma	Urine	male	interm risk
189036	64	Prostate Carcinoma	Urine	male	low risk
189037	56	Prostate Carcinoma	Urine	male	low risk
189038	67	Prostate Carcinoma	Urine	male	high risk
189054	79	Prostate Carcinoma	Urine	male	interm risk
189058	69	Prostate Carcinoma	Urine	male	low risk
189060	52	Prostate Carcinoma	Urine	male	low risk
189062	58	Prostate Carcinoma	Urine	male	low risk
189069	70	Prostate Carcinoma	Urine	male	interm risk
189070	78	Prostate Carcinoma	Urine	male	interm risk
189080	59	Prostate Carcinoma	Urine	male	low risk
189081	54	Prostate Carcinoma	Urine	male	low risk
189083	73	Prostate Carcinoma	Urine	male	interm risk
189085	63	Prostate Carcinoma	Urine	male	low risk
189087	72	Prostate Carcinoma	Urine	male	interm risk
189088	40	Prostate Carcinoma	Urine	male	low risk
189089	57	Prostate Carcinoma	Urine	male	low risk
189093	72	Prostate Carcinoma	Urine	male	interm risk
189094	67	Prostate Carcinoma	Urine	male	interm risk
189095	72	Prostate Carcinoma	Urine	male	low risk
189096	66	Prostate Carcinoma	Urine	male	low risk
189099	66	Prostate Carcinoma	Urine	male	interm risk
189100	78	Prostate Carcinoma	Urine	male	interm risk
189101	74	Prostate Carcinoma	Urine	male	high risk
189102	70	Prostate Carcinoma	Urine	male	low risk
189106	60	Prostate Carcinoma	Urine	male	interm risk
189111	75	Prostate Carcinoma	Urine	male	interm risk
189112	71	Prostate Carcinoma	Urine	male	interm risk
189113	59	Prostate Carcinoma	Urine	male	interm risk
189114	67	Prostate Carcinoma	Urine	male	interm risk
189115	54	Prostate Carcinoma	Urine	male	low risk
189116	77	Prostate Carcinoma	Urine	male	low risk
189120	71	Prostate Carcinoma	Urine	male	low risk
189121	53	Prostate Carcinoma	Urine	male	low risk
189122	66	Prostate Carcinoma	Urine	male	low risk
189123	56	Prostate Carcinoma	Urine	male	low risk
189125	57	Prostate Carcinoma	Urine	male	low risk
189126	51	Prostate Carcinoma	Urine	male	high risk
189127	51	Prostate Carcinoma	Urine	male	low risk
189128	55	Prostate Carcinoma	Urine	male	low risk
189129	63	Prostate Carcinoma	Urine	male	low risk

189131	66	Prostate Carcinoma	Urine	male	low risk
189133	58	Prostate Carcinoma	Urine	male	low risk
189134	66	Prostate Carcinoma	Urine	male	low risk
189135	73	Prostate Carcinoma	Urine	male	interm risk
189136	44	Prostate Carcinoma	Urine	male	low risk
189138	68	Prostate Carcinoma	Urine	male	low risk
189139	45	Prostate Carcinoma	Urine	male	interm risk
189140	74	Prostate Carcinoma	Urine	male	low risk
189141	75	Prostate Carcinoma	Urine	male	high risk
189142	65	Prostate Carcinoma	Urine	male	low risk
189143	72	Prostate Carcinoma	Urine	male	high risk
189144	55	Prostate Carcinoma	Urine	male	interm risk
189146	72	Prostate Carcinoma	Urine	male	interm risk
189147	79	Prostate Carcinoma	Urine	male	interm risk
189148	61	Prostate Carcinoma	Urine	male	low risk
189149	67	Prostate Carcinoma	Urine	male	high risk
189151	68	Prostate Carcinoma	Urine	male	low risk
189152	66	Prostate Carcinoma	Urine	male	low risk
189154	52	Prostate Carcinoma	Urine	male	low risk
189155	72	Prostate Carcinoma	Urine	male	low risk
189156	73	Prostate Carcinoma	Urine	male	interm risk
189157	49	Prostate Carcinoma	Urine	male	low risk
189240	67	Prostate Carcinoma	Urine	male	interm risk
189248	65	Prostate Carcinoma	Urine	male	high risk
189259	64	Prostate Carcinoma	Urine	male	low risk
189260	54	Prostate Carcinoma	Urine	male	interm risk
189261	69	Prostate Carcinoma	Urine	male	interm risk
189263	84	Prostate Carcinoma	Urine	male	high risk
189264	73	Prostate Carcinoma	Urine	male	high risk
189265	69	Prostate Carcinoma	Urine	male	interm risk
189267	75	Prostate Carcinoma	Urine	male	low risk
189268	52	Prostate Carcinoma	Urine	male	interm risk
189269	61	Prostate Carcinoma	Urine	male	low risk
189271	68	Prostate Carcinoma	Urine	male	low risk
189272	75	Prostate Carcinoma	Urine	male	low risk
189277	68	Prostate Carcinoma	Urine	male	low risk
189278	69	Prostate Carcinoma	Urine	male	low risk
189280	50	Prostate Carcinoma	Urine	male	low risk
189282	67	Prostate Carcinoma	Urine	male	low risk
189283	72	Prostate Carcinoma	Urine	male	high risk
189285	60	Prostate Carcinoma	Urine	male	low risk
189292	58	Prostate Carcinoma	Urine	male	low risk
189293	69	Prostate Carcinoma	Urine	male	interm risk
189294	53	Prostate Carcinoma	Urine	male	interm risk
189295	63	Prostate Carcinoma	Urine	male	low risk
189296	71	Prostate Carcinoma	Urine	male	high risk
189297	66	Prostate Carcinoma	Urine	male	high risk
189298	68	Prostate Carcinoma	Urine	male	low risk
189299	60	Prostate Carcinoma	Urine	male	low risk

189300	64	Prostate Carcinoma	Urine	male	low risk
189304	72	Prostate Carcinoma	Urine	male	interm risk
189305	62	Prostate Carcinoma	Urine	male	low risk
189306	60	Prostate Carcinoma	Urine	male	low risk
189308	63	Prostate Carcinoma	Urine	male	low risk
189309	70	Prostate Carcinoma	Urine	male	low risk
189311	66	Prostate Carcinoma	Urine	male	low risk
189312	61	Prostate Carcinoma	Urine	male	low risk
189313	54	Prostate Carcinoma	Urine	male	interm risk
189314	74	Prostate Carcinoma	Urine	male	high risk
189350	67	Prostate Carcinoma	Urine	male	interm risk
189351	66	Prostate Carcinoma	Urine	male	low risk
189352	70	Prostate Carcinoma	Urine	male	interm risk
189353	72	Prostate Carcinoma	Urine	male	high risk
189354	62	Prostate Carcinoma	Urine	male	low risk
189355	50	Prostate Carcinoma	Urine	male	interm risk
189356	59	Prostate Carcinoma	Urine	male	low risk
189358	65	Prostate Carcinoma	Urine	male	low risk
189359	59	Prostate Carcinoma	Urine	male	low risk
189360	70	Prostate Carcinoma	Urine	male	low risk
189361	54	Prostate Carcinoma	Urine	male	low risk
189363	68	Prostate Carcinoma	Urine	male	low risk
189364	66	Prostate Carcinoma	Urine	male	low risk
189365	63	Prostate Carcinoma	Urine	male	low risk
189366	65	Prostate Carcinoma	Urine	male	low risk
189367	67	Prostate Carcinoma	Urine	male	low risk
189368	51	Prostate Carcinoma	Urine	male	low risk
189369	65	Prostate Carcinoma	Urine	male	interm risk
189370	61	Prostate Carcinoma	Urine	male	interm risk
189371	67	Prostate Carcinoma	Urine	male	high risk
189372	67	Prostate Carcinoma	Urine	male	low risk
189373	61	Prostate Carcinoma	Urine	male	low risk
189374	82	Prostate Carcinoma	Urine	male	high risk
189375	70	Prostate Carcinoma	Urine	male	low risk
189392	68	Prostate Carcinoma	Urine	male	low risk
189393	52	Prostate Carcinoma	Urine	male	high risk
189394	71	Prostate Carcinoma	Urine	male	interm risk
189395		Prostate Carcinoma	Urine	male	interm risk
189396	51	Prostate Carcinoma	Urine	male	low risk
189397	63	Prostate Carcinoma	Urine	male	low risk
189398	67	Prostate Carcinoma	Urine	male	interm risk
189399	52	Prostate Carcinoma	Urine	male	interm risk
189408	72	Prostate Carcinoma	Urine	male	low risk
189411	62	Prostate Carcinoma	Urine	male	low risk
189415	48	Prostate Carcinoma	Urine	male	low risk
189417	77	Prostate Carcinoma	Urine	male	low risk
189418	75	Prostate Carcinoma	Urine	male	low risk
189419	62	Prostate Carcinoma	Urine	male	interm risk
189449	59	Prostate Carcinoma	Urine	male	low risk
189450	51	Prostate Carcinoma	Urine	male	low risk

Prostate volume at biopsy (ccm3)	PSA at biopsy (ng/ml)	Digital Rectal Examination (0=normal; 1=suspicious)
60	3,2	0
	8,31	
25	2,3	0
53	9,9	0
52	6,7	1
35	13,15	0
66	10,85	0
	2,24	1
	8,45	0
20	4,42	0
52	20,88	0
40	15,41	0
42	4,2	0
35	3,24	0
45	7,47	0
120	4,13	
45	17,89	0
32	4,92	
49	1,97	0
35	4,47	0
20	3,3	0
70	7,35	1
	4,65	0
40	2,23	0
100	8,06	0
40	13,9	0
29	4,61	0
25	3,44	
25	6,1	0
30	3,16	0
42	26,66	0
	4,24	
25	5,5	0
63	11,34	0
60	8,64	0
55	4,28	0
21	4,29	
40	4,06	0
30	8,7	0
40	9,37	0
20	4,21	0
85	8,25	0
35	7,2	0
40	3,42	0
30	15,2	0
25	4	0
35	2,98	1
50	2,93	0

75	5,2	0
50	3,15	0
50	6,94	
20	3,38	0
25	1,67	0
30	4,65	
22	4,45	
35	4,99	0
40	5,9	0
25	2,52	0
40	18,09	1
85	5,67	0
90	15,02	
20	13,92	
60	4,61	0
50	20,75	0
40	3,05	
45	9,28	1
45	4,38	0
65	7,17	1
45	9,4	0
30	2,82	1
50	10,99	
43	3,73	0
33	3,92	1
30	6,51	
30	3,39	1
50	23,64	1
	9,66	0
77	7,2	0
49	9,54	0
32	3,55	1
35	4,1	0
80	6,33	0
50	4,2	
50	7,4	1
65	4,95	0
	4,79	
60	3,85	0
70	21,33	0
65	4,46	0
19	1,97	0
50	13,27	1
30	10,5	0
71	4,1	0
65	11,44	1
80	19,4	1
	5,7	
20	2,16	0

28	3,8	1
	8,22	
32	5,57	0
25	2,51	
65	8,24	
40	5,55	
20	4,2	0
40	2,71	0
46	5,91	
100	34,74	1
	12,52	0
35	3,13	
27	3,63	0
50	9,52	0
25	5,5	0
	6,4	0
28	3,21	0
35	3,27	0
35	5,36	
30	3,18	0
30	4,64	1
54	5,42	0
69	6,78	0
59	5,29	0
29	2,61	
30	6,47	
30	3,72	0
30	15	
45	3,37	0
65	10,7	
35	8,19	0
50	4,24	
55	19,18	0
45	3,19	0
	5,24	
25	2,85	
35	4,09	
60	11,05	0
36	3,1	0
30	4,62	
23	2,83	
35	3,73	0
91	6,05	
30	4,46	0
30	2,14	1
65	9,33	0
52	7,57	
57	12,39	
45	5,85	0
30	4,27	0

Biopsy_Gleason_Score (ROC)	Biopsy Gleason Score	Biopsy Gleason Pattern 1
0	6	3
1	7	3
0	6	3
0	6	3
1	7	3
0	6	3
0	6	3
1	7	3
0	6	3
0	6	3
1	7	3
0	6	3
0	6	3
0	6	3
0	6	3
1	7	3
1	7	3
0	6	3
0	6	3
1	7	3
0	6	3
1	7	3
0	6	3
0	6	3
1	7	3
1	7	3
0	6	3
0	6	3
1	7	3
1	7	3
1	8	3
0	6	3
1	7	3
0	6	3
1	7	4
1	7	3
1	7	3
0	6	3
0	6	3
0	6	3
0	6	3
0	6	3
0	6	3
0	6	3
1	8	5
0	6	3
0	6	3
0	6	3

0	6	3
0	6	3
0	6	3
1	7	3
0	6	3
0	6	3
1	7	3
0	6	3
1	9	4
0	6	3
1	8	3
1	7	3
0	6	3
1	7	4
0	6	3
0	6	3
0	6	3
0	6	3
0	6	3
0	6	3
1	7	3
0	6	3
1	7	3
1	8	3
0	6	3
1	7	3
1	7	3
1	7	3
1	9	5
1	7	4
0	6	3
1	7	4
0	6	3
0	6	3
0	6	3
0	6	3
0	6	3
0	6	3
0	6	3
0	6	3
0	6	3
0	6	3
0	6	3
0	6	3
0	6	3
1	8	4
1	9	4
0	6	3
0	6	3

0	6	3
1	7	3
0	6	3
0	6	3
0	6	3
0	6	3
0	6	3
0	6	3
1	7	3
1	9	4
0	6	3
0	6	3
1	7	3
1	9	4
0	6	3
1	7	3
0	6	3
0	6	3
0	6	3
0	6	3
0	6	3
0	6	3
0	6	3
0	6	3
0	6	3
0	6	3
0	6	3
0	6	3
1	7	3
1	8	4
0	6	3
0	6	3
1	8	3
0	6	3
0	6	3
1	8	3
1	7	3
0	6	3
0	6	3
0	6	3
1	7	3
1	7	3
0	6	3
0	6	3
0	6	3
0	6	3
0	6	3
0	6	3
0	6	3
0	6	3

Biopsy Gleason Pattern 2	Histology_Results	Percentage of free PSA (% at biopsy)	Prostatic capsule at the biopsy
3	Adenocarcinoma		
4	adenocarcinoma	12	
3	adenocarcinoma	10,4	intact
3	adenocarcinoma		
4	adenocarcinoma	55,3	intact
3	adenocarcinoma	16	
3	adenocarcinoma	27,6	intact
4	adenocarcinoma	15,2	intact
3	adenocarcinoma	9,8	
3	adenocarcinoma	27,4	intact
4	adenocarcinoma	7,5	intact
3	adenocarcinoma	14,7	
3	adenocarcinoma	24	intact
3	adenocarcinoma	13,9	intact
3	adenocarcinoma	5,8	intact
4	adenocarcinoma	58,84	
4	adenocarcinoma	12,97	intact
3	adenocarcinoma	7,7	intact
3	adenocarcinoma	13,2	intact
4	adenocarcinoma	19	intact
3	adenocarcinoma	9	intact
4	adenocarcinoma	20,6	intact
3	adenocarcinoma	8	
3	adenocarcinoma	13	intact
4	adenocarcinoma	28	intact
4	adenocarcinoma		
3	adenocarcinoma	10,6	intact
3	adenocarcinoma	18,7	
4	adenocarcinoma	20,1	intact
4	adenocarcinoma	12,2	intact
5	adenocarcinoma	10,3	intact
3	adenocarcinoma	18,9	
4	adenocarcinoma	7,4	intact
3	adenocarcinoma	25,5	
3	adenocarcinoma	21,1	intact
4	adenocarcinoma	16,59	intact
4	adenocarcinoma	7,2	intact
3	adenocarcinoma	17,6	intact
3	adenocarcinoma	15	intact
3	adenocarcinoma	10	
3	adenocarcinoma	17,81	intact
3	adenocarcinoma		intact
3	adenocarcinoma	7,2	intact
3	adenocarcinoma	14,04	intact
3	adenocarcinoma	12	intact
3	adenocarcinoma	14	intact
3	adenocarcinoma	25,5	intact
3	adenocarcinoma	15	intact

3	adenocarcinoma	19	
3	adenocarcinoma	14,8	intact
3	adenocarcinoma	12,8	
4	adenocarcinoma	9,1	
3	adenocarcinoma	10,2	intact
3	adenocarcinoma	20	intact
4	adenocarcinoma	10	
3	adenocarcinoma	15,4	intact
5	adenocarcinoma	20	intact
3	adenocarcinoma	11,11	intact
5	adenocarcinoma	8,96	intact
4	adenocarcinoma	34,39	intact
3	adenocarcinoma	20,37	intact
3	adenocarcinoma	31,54	
3	adenocarcinoma	23,64	intact
3	adenocarcinoma	10,46	[illegible] cancer
3	adenocarcinoma	11,8	
3	adenocarcinoma	8	intact
3	adenocarcinoma	20,09	intact
3	adenocarcinoma		intact
4	adenocarcinoma	9	intact
3	adenocarcinoma	16,67	
4	adenocarcinoma	28,57	
5	adenocarcinoma	10,99	
3	adenocarcinoma	21,17	intact
4	adenocarcinoma	15,36	
4	adenocarcinoma	12	intact
4	adenocarcinoma	20,7	
4	adenocarcinoma	22,67	
3	adenocarcinoma	13,67	intact
3	adenocarcinoma	13,2	intact
3	adenocarcinoma	11,55	intact
3	adenocarcinoma	30,75	intact
3	adenocarcinoma	18,17	
3	adenocarcinoma	20	
3	adenocarcinoma		intact
3	adenocarcinoma	29,9	intact
3	adenocarcinoma	22	
3	adenocarcinoma	20,52	intact
3	adenocarcinoma	9,33	intact
3	adenocarcinoma	28,9	intact
3	adenocarcinoma	7,11	intact
3	adenocarcinoma	14,39	intact
3	adenocarcinoma	13	intact
3	adenocarcinoma		
4	adenocarcinoma	21,2	
5	adenocarcinoma		[illegible] cancer
3	adenocarcinoma	16,3	
3	adenocarcinoma	9,47	intact

3	adenocarcinoma	14,5	intact
4	adenocarcinoma	7,18	
3	adenocarcinoma	30,3	
3	adenocarcinoma	14,7	intact
3	adenocarcinoma	16,8	
3	adenocarcinoma	28,5	
3	adenocarcinoma	8	
3	adenocarcinoma	17,5	
4	adenocarcinoma	12	
5	adenocarcinoma		
3	adenocarcinoma		intact
3	adenocarcinoma	12,5	intact
4	adenocarcinoma	17,7	intact
5	adenocarcinoma	8,2	
3	adenocarcinoma	22	intact
4	adenocarcinoma	6,3	intact
3	adenocarcinoma	8,1	intact
3	adenocarcinoma	19,3	intact
3	adenocarcinoma	12,7	intact
3	adenocarcinoma	11,2	intact
3	adenocarcinoma	15,1	
3	adenocarcinoma	19,4	intact
3	adenocarcinoma	15	
3	adenocarcinoma	24	
3	adenocarcinoma	14,6	intact
3	adenocarcinoma	12,67	intact
3	adenocarcinoma	8,3	intact
3	adenocarcinoma	5	
4	adenocarcinoma	15,4	
4	adenocarcinoma		intact
3	adenocarcinoma	18,6	
3	adenocarcinoma	20,28	intact
5	Adenocarcinoma	32,2	
3	adenocarcinoma	12	intact
3	adenocarcinoma	10,5	intact
5	adenocarcinoma	11,58	intact
4	adenocarcinoma	21,03	intact
3		23,8	intact
3	adenocarcinoma	15,8	intact
3	adenocarcinoma	18,4	intact
4	adenocarcinoma	8	intact
4	adenocarcinoma	14,7	intact
3	adenocarcinoma	23	
3	adenocarcinoma	24	intact
3	adenocarcinoma	13,6	intact
3	adenocarcinoma	9,3	intact
3	adenocarcinoma	22,85	intact
3	adenocarcinoma	28	intact
3	adenocarcinoma	23	
3	adenocarcinoma	17,33	

Number of biopsies	Number of positive biopsies (%)
0	6,67
1	25
0	6,67
0	33,33
0	66,67
0	
1	26,67
0	13,33
0	
0	20
1	26,67
0	13,33
0	20
1	14,29
1	13,33
0	13,33
1	6,67
1	26,67
0	33,33
0	20
0	13,33
0	20
0	40
0	13,33
0	26,67
0	66,67
0	40
0	10
0	46,67
0	46,67
1	26,67
0	20
0	28,57
1	7,69
1	33,33
0	20
1	20
0	
0	26,67
0	6,67
1	46,67
0	14,29
1	33,33
0	20
0	66,67
0	60
1	13,33
0	13,33

0	10
0	21,43
1	33,33
1	13,33
0	6,67
0	33,33
0	20
0	20
0	33,33
0	13,33
0	26,67
0	21,43
0	13,33
1	33,33
0	40
1	13,33
0	6,67
1	20
1	6,67
0	20
0	13,33
0	33,33
0	26,67
0	53,33
0	53,33
1	33,33
1	60
0	10
0	38,46
0	60
1	26,67
0	46,67
1	10
1	6,67
0	13,33
0	33,33
0	23,08
1	20
0	13,33
1	6,67
1	6,67
0	6,67
1	13,33
0	40
0	6,67
0	6,67
0	53,33
0	
0	13,33

0	
0	85,71
0	18,75
0	13,33
0	28,57
0	13,33
0	
0	33,33
1	70
0	66,67
1	13,33
0	16,67
0	6,67
1	20
0	13,33
1	26,67
1	6,67
0	26,67
1	6,67
1	6,67
0	13,33
1	13,33
0	20
0	6,67
0	6,67
1	13,33
0	6,67
0	30,77
0	20
1	66,67
0	26,67
1	13,33
0	94,12
1	20
0	20
0	20
0	14,29
0	
0	13,33
0	16,67
0	20
0	8,33
0	33,33
0	13,33
0	8,33
1	13,33
1	13,33
0	26,67
0	20
0	10

Urine Creatinine (mmol/l)	Urine Total Protein (g/l)	Radical Prostatectomy: Gleason Score
5,4	0,03	7
9,3	0,03	7
15	0,03	
9,8	0,03	
18,6	0,13	
9,1	0,03	
5	0,03	6
10,7	0,05	6
4,2	0,03	
12,6	0,08	7
10,7	0,03	8
11,8	0,03	
7,6	0,03	7
19,4	2,53	7
1,2	0,03	7
22,7	0,13	
8,1	0,03	
14,6	0,05	6
3	0,03	6
7,8	0,05	7
8,2	0,08	7
10,8	0,03	7
22,9	0,13	?
		5
8,8	0,09	
3,9	0,06	
13,7	0,03	
6,2	0,03	7
20	0,09	7
7,6	0,05	7
17	5,88	
18,3	0,07	
20	0,08	
6,5	0,05	
22,4	0,06	9
7,7	0,06	7
8,7	0,06	7
5,2	0,05	6
11,8	0,06	
6,9	0,03	7
10,3	0,03	7
2,3	0,03	
13,1	0,05	7
3,1	0,03	6
10,5	0,11	9
		7
19	0,07	5
7,8	0,03	6

10,4	0,03	
9,7	0,03	6
6,4	0,05	7
9	0,03	7
3,6	0,03	6
16,7	0,2	
20	0,08	
11,7	0,03	
10,5	0,03	
13,8	0,05	7
9,6	0,05	9
4	0,03	7
11,7	0,06	
11,7	0,03	
2	0,03	7
15,5	0,05	8
13,8	0,03	
21,1	0,16	7
10,9	0,03	7
9,5	0,14	
20,6	0,05	
13,4	0,05	7
6,9	0,11	
5,8	0,03	
21	0,09	7
9	0,03	
17,1	0,08	7
7,6	0,08	
16,5	0,1	
2	0,03	7
5,7	0,03	7
3,6	0,03	7
7,8	0,03	6
15	0,05	5
8,4	0,03	
6,7	0,03	7
5,8	0,03	6
2,7	0,03	7
12,3	0,03	7
13,9	0,05	7
1,8	0,03	5
3,7	0,03	7
11,3	0,03	
7,9	0,03	7
		7
5	0,03	5
11,2	0,07	7

6,8	0,72	7
12,1	0,05	
		7
2,4		5
		7
		7
5,6	0,1	
16	0,05	6
1,5	0,03	7
8,3	0,08	
		7
		6
4,2	0,06	7
16,6	0,12	9
6	0,37	6
4,9	0,03	
7,6	0,03	7
2,3	0,03	
5,7	0,03	7
5,3	0,03	5
3,5	0,03	6
9	0,05	
7,6	0,03	6
4	0,03	
3	0,03	6
11,6	0,03	
10,8	0,03	7
2,6	0,03	
6,2	0,03	
18,5		
15,8		
10,6	0,03	7
2,1	0,03	
11,5	0,03	5
4,3	0,03	
11,8	0,03	
5,5	0,35	7
3,7	0,03	6
10,6	0,06	7
2,8	0,03	7
15		7
7,3	0,05	7
22,5	0,1	
9,5	0,05	5
2,1	0,03	7
2,2	0,06	
3,9	0,03	
		7
6,4	0,82	7
6,6	0,03	6

Radical Prostatectomy: Gleason Pattern 1	Radical Prostatectomy: Gleason Pattern 2	Free PSA (%) at Radical Prostatectomy
4	3	18,3
3	4	9,7
3	3	22,5
3	3	21,2
3	4	27,4
5	3	
3	4	21,7
3	4	13,9
3	4	9,8
3	3	7
3	3	14,3
4	3	117,7
3	4	13
4	3	17,1
		10,5
3	2	10
3	4	
4	3	20,1
3	4	
4	5	19,3
4	3	16,5
4	3	7,67
3	3	
3	4	8,36
3	4	
3	4	
3	3	14,4
5	4	10,7
4	3	23,06
3	2	21,7
3	3	21,46

3	3	14,7
3	4	8,2
4	3	10,23
3	3	17,68
3	4	19,77
4	5	9,18
4	3	32,51
3	4	
4	4	10
3	4	8
3	4	20
4	3	11,31
3	4	
		15,36
4	3	15,6
3	4	33,82
3	4	16
4	3	11,55
3	3	
3	2	25,36
3	4	
3	3	35,33
3	4	22
4	3	10
4	3	
3	2	
3	4	9,2
3	4	13
4	3	15,8
2	3	
4	3	8,7

3	4	
3	4	21,7
3	2	17,7
3	4	16,8
3	4	28,5
3	3	
3	4	12,2
3	4	13,62
3	3	12,5
3	4	31,3
5	4	8,2
3	3	27,3
3	4	8,1
3	4	12,72
3	2	11,2
3	3	9,9
3	3	22,05
3	3	13,3
3	4	9,4
3	4	26,45
3	2	12
3	4	21,03
3	3	27,84
3	4	15,24
3	4	18,4
3	4	7,5
3	4	14,7
3	2	19
3	4	13,6
3	4	28
3	4	23
3	3	17,33

Prostate Weight at Radical Prostatectomy (g)	Organ confined / non organ confined	Pathologic T category (TNM staging system)
41	organ-confined	2c
50	organ-confined	2c
65	organ-confined	2c
33	organ-confined	2c
16	non-organ-confined	2c
42	non-organ-confined	3a
46		2c
25	non-organ-confined	3a
40	organ-confined	2a
30	organ-confined	2b
41	organ-confined	2c
36	organ-confined	3a
50	no information available	2c
60	organ-confined	2c
	organ-confined	2c
37	organ-confined	2c
38	organ-confined	2c
30	organ-confined	3a
	organ-confined	2c
52	organ-confined	2c
36	organ-confined	3b
38	organ-confined	2c
	organ-confined	2c
48	organ-confined	2c
36	organ-confined	2c
36	non-organ-confined	3a
	organ-confined	2c
44	non-organ-confined	3b
33	non-organ-confined	2c
34	organ-confined	2c
55	organ-confined	2c

47	organ-confined	2c
46	non-organ-confined	3a
29	non-organ-confined	3a
37	organ-confined	2a
35	organ-confined	2c
53	non-organ-confined	3b
	organ-confined	2c
72	organ-confined	2c
60	non-organ-confined	3b
47	organ-confined	2c
54	organ-confined	2c
	organ-confined	2c
35	organ-confined	2c
35	non-organ-confined	3a
86	organ-confined	2c
80	organ-confined	2c
41	non-organ-confined	3a
		?
100	organ-confined	2c
56	organ-confined	2c
64	organ-confined	2c
38	organ-confined	2c
57	organ-confined	2c
59	non-organ-confined	3a
75	organ-confined	2c
33	organ-confined	2c
30	organ-confined	2c
70	organ-confined	2c
50	organ-confined	2c
18	organ-confined	2c

	non-organ-confined	2c
30	organ-confined	2c
30	organ-confined	2a
43	non-organ-confined	2c
	organ-confined	2c
40	organ-confined	2c
46	organ-confined	2c
	non-organ-confined	2c
	organ-confined	2b
39	organ-confined	2c
56	organ-confined	3a
23	organ-confined	2c
30	organ-confined	2c
45	organ-confined	2c
41	organ-confined	2a
25	organ-confined	2c
59	non-organ-confined	2c
28	organ-confined	2c
25	organ-confined	2c
	organ-confined	2c
43	organ-confined	2c
48	non-organ-confined	2c
84		
40	organ-confined	2c
38	organ-confined	2c
35	organ-confined	2c
35	organ-confined	2c
54	organ-confined	2c
30	organ-confined	2c
55	organ-confined	3a
45	organ-confined	3a
	organ-confined	2a

pathologic M category (TNM staging system)	pathologic N category (TNM staging system)	PSA before Radical Prostatectomy [ng/ml]
1	1	4,099
1	0	7,15
1	0	9,17
1	1	2,41
1	1	4,42
1	1	20,88
1	1	3,89
1	1	3,2
1	1	6,88
1	1	4,92
1	1	2,05
1	1	4,82
1	1	3,5
1	0	6,51
1	1	4
1	0	2,319
1	1	
1	0	5,31
1	1	1,14
1	0	8,6
1	1	4,28
1	0	3,52
1	1	
1	1	6,1
1	1	
1	1	
1	1	3,42
1	1	18,78
1	1	3,86
1	1	3,31
1	1	2,47

1	1	3,97
1	1	7,16
1	1	4,3
		1,81
		10,44
1	1	2,58
1	1	8,61
1	1	4,46
1	1	4,6
1	1	20
1	0	9,28
1	1	4,38
1	1	3,27
1	1	3,92
		6,51
1	1	3,59
1	0	6,21
1	0	11
1	1	3,55
1	1	5,6
1	1	7,4
1	1	3,86
1	1	4,79
1	1	3,8
1	1	22
1	1	4,46
1	1	1,74
1	1	10,48
1	0	11,09
1	0	
1	1	3,21

1	0	
1	1	6,27
1	1	2,32
1	0	8,14
1	1	5,55
1	1	
1	0	4,92
1	0	10,6
1	1	3,13
1	1	1,57
1	0	9,52
1	1	4,83
1	1	3,21
1	1	5,66
1	1	3,18
1	1	7,79
1		5,07
1	1	2,93
1	1	4,15
1	1	3,63
1	1	3,19
1	0	4,09
		23,85
1	0	3,28
1	1	4,62
1	0	3,6
1	0	3,73
1	1	4,41
1	1	2,11
1	0	12,39
1	1	5,85
1	1	4,27
