# Supplementary material for: Validation of diagnostic nomograms based on CE–MS urinary biomarkers to detect clinically significant prostate cancer
Source: World J Urol. 2022 Jul 16;40(9):2195–203. doi: 10.1007/s00345-022-04077-1 (PMC9427869; doi:10.1007/s00345-022-04077-1)
Supplement: Supplementary file 2 — Supplementary file2 Supplementary Table S2: List of scoring data for 147 patients with PCa, including the SVM-based and the nomogram scoring data (PDF 462 KB) [file 345_2022_4077_MOESM2_ESM.pdf]

CE-MS_Analysis ID	PSA at biopsy [ng/ml]	PSA density	Age at the time of diagnosis
189008	3,2	0,053333333	66
189022	8,31	NA	72
189023	2,3	0,092	66
189024	9,9	0,186792453	77
189025	6,7	0,128846154	73
189026	13,15	0,375714286	61
189027	10,85	0,164393939	78
189033	2,24	NA	59
189036	8,45	NA	64
189037	4,42	0,221	56
189038	20,88	0,401538462	67
189054	15,41	0,38525	79
189058	4,2	0,1	69
189060	3,24	0,092571429	52
189062	7,47	0,166	58
189069	4,13	0,034416667	70
189070	17,89	0,397555556	78
189080	4,92	0,15375	59
189081	1,97	0,040204082	54
189083	4,47	0,127714286	73
189085	3,3	0,165	63
189087	7,35	0,105	72
189088	4,65	NA	40
189089	2,23	0,05575	57
189093	8,06	0,0806	72
189094	13,9	0,3475	67
189095	4,61	0,158965517	72
189096	3,44	0,1376	66
189099	6,1	0,244	66
189100	3,16	0,105333333	78
189101	26,66	0,634761905	74
189102	4,24	NA	70
189106	5,5	0,22	60
189111	11,34	0,18	75
189112	8,64	0,144	71
189113	4,28	0,077818182	59
189114	4,29	0,204285714	67
189115	4,06	0,1015	54
189116	8,7	0,29	77
189120	9,37	0,23425	71
189121	4,21	0,2105	53
189122	8,25	0,097058824	66
189123	7,2	0,205714286	56
189125	3,42	0,0855	57
189126	15,2	0,506666667	51
189127	4	0,16	51
189128	2,98	0,085142857	55
189129	2,93	0,0586	63

189131	5,2	0,069333333	66
189133	3,15	0,063	58
189134	6,94	0,1388	66
189135	3,38	0,169	73
189136	1,67	0,0668	44
189138	4,65	0,155	68
189139	4,45	0,202272727	45
189140	4,99	0,142571429	74
189141	5,9	0,1475	75
189142	2,52	0,1008	65
189143	18,09	0,45225	72
189144	5,67	0,066705882	55
189146	15,02	0,166888889	72
189147	13,92	0,696	79
189148	4,61	0,076833333	61
189149	20,75	0,415	67
189151	3,05	0,07625	68
189152	9,28	0,206222222	66
189154	4,38	0,097333333	52
189155	7,17	0,110307692	72
189156	9,4	0,208888889	73
189157	2,82	0,094	49
189240	10,99	0,2198	67
189248	3,73	0,086744186	65
189259	3,92	0,118787879	64
189260	6,51	0,217	54
189261	3,39	0,113	69
189263	23,64	0,4728	84
189264	9,66	NA	73
189265	7,2	0,093506494	69
189267	9,54	0,194693878	75
189268	3,55	0,1109375	52
189269	4,1	0,117142857	61
189271	6,33	0,079125	68
189272	4,2	0,084	75
189277	7,4	0,148	68
189278	4,95	0,076153846	69
189280	4,79	NA	50
189282	3,85	0,064166667	67
189283	21,33	0,304714286	72
189285	4,46	0,068615385	60
189292	1,97	0,103684211	58
189293	13,27	0,2654	69
189294	10,5	0,35	53
189295	4,1	0,057746479	63
189296	11,44	0,176	71
189297	19,4	0,2425	66
189298	5,7	NA	68
189299	2,16	0,108	60
189300	3,8	0,135714286	64

189304	8,22	NA	72
189305	5,57	0,1740625	62
189306	2,51	0,1004	60
189308	8,24	0,126769231	63
189309	5,55	0,13875	70
189311	4,2	0,21	66
189312	2,71	0,06775	61
189313	5,91	0,128478261	54
189314	34,74	0,3474	74
189350	12,52	NA	67
189351	3,13	0,089428571	66
189352	3,63	0,134444444	70
189353	9,52	0,1904	72
189354	5,5	0,22	62
189355	6,4	NA	50
189356	3,21	0,114642857	59
189358	3,27	0,093428571	65
189359	5,36	0,153142857	59
189360	3,18	0,106	70
189361	4,64	0,154666667	54
189363	5,42	0,10037037	68
189364	6,78	0,09826087	66
189365	5,29	0,089661017	63
189366	2,61	0,09	65
189367	6,47	0,215666667	67
189368	3,72	0,124	51
189369	15	0,5	65
189370	3,37	0,074888889	61
189371	10,7	0,164615385	67
189372	8,19	0,234	67
189373	4,24	0,0848	61
189374	19,18	0,348727273	82
189375	3,19	0,070888889	70
189392	5,24	NA	68
189393	2,85	0,114	52
189394	4,09	0,116857143	71
189395	11,05	0,184166667	
189396	3,1	0,086111111	51
189397	4,62	0,154	63
189398	2,83	0,123043478	67
189399	3,73	0,106571429	52
189408	6,05	0,066483516	72
189411	4,46	0,148666667	62
189415	2,14	0,071333333	48
189417	9,33	0,143538462	77
189418	7,57	0,145576923	75
189419	12,39	0,217368421	62
189449	5,85	0,13	59
189450	4,27	0,142333333	51

19-BM score (based on SVM)	ERSPC-3/4	DN: PSA-AGE-19BM
-0,47	0,03	0,13890345
0,202		0,39640436
-1,28	0,02	0,0477535
-0,84	0,11	0,1659185
1,401	0,34	0,7613303
-0,21	0,16	0,26713063
-0,099	0,02	0,37523779
1,309	0,1	0,59012089
-0,66	0,09	0,13870756
-0,13	0,04	0,1723871
0,277	0,04	0,57404808
0,852	0,19	0,75061114
0,05	0,04	0,27623452
0,26	0,01	0,22831223
0,95	0,02	0,53935502
-0,039		0,25706374
1,102	0,03	0,8273272
0,81		0,46005623
0,04	0,01	0,17654163
0,182	0,04	0,34273436
-0,37	0,03	0,14597288
1,375	0,37	0,75674005
-0,76	0,04	0,05323282
0,16	0,02	0,21865174
0,399	0,09	0,45893991
-0,022	0,17	0,36957559
-0,01	0,04	0,28184795
-0,4		0,15268824
1,768	0,06	0,80668395
0,192	0,03	0,36040159
-1,019	0,04	0,28183906
-0,9		0,09631762
1,683	0,05	0,7510325
0,452	0,02	0,5478263
1,338	0,02	0,75644412
0,669	0,04	0,40307701
1,141		0,61906941
0,807	0,04	
-1,27	0,1	0,09290687
-0,26	0,11	0,26493388
-1,38	0,01	0,03272481
0,46	0,09	0,44013415
-0,72	0,01	0,09863295
-0,82	0,03	0,07245486
1,271	0,19	0,70386193
-0,17	0,03	0,1428316
-0,6	0,03	0,08861422
-1,16	0,02	0,05338325

-0,91	0,05	0,09038884
-0,73	0,03	0,08214698
-1,2		0,06896153
1,703	0,01	0,79818746
-0,84	0,01	0,04504583
-0,48		0,15530812
1,546		0,60442148
-0,64	0,05	0,15164816
0,655	0,06	0,535645
-0,83	0,02	0,0840039
1,07	0,67	0,79632646
1,296	0,06	0,6080962
1,391		0,83475731
0,328		0,57230915
-1,13	0,04	0,05787332
1,015	0,04	0,7867679
-0,29		0,17817038
-1,24	0,07	0,07470517
-1,08	0,01	0,04778647
-1,01	0,36	0,10375443
1,915	0,11	0,88389506
-0,4	0,13	0,09650929
0,067		0,35727072
0,338	0,03	0,32966017
-0,42	0,19	0,14562496
0,291		0,28479786
0,373	0,03	0,36173162
-0,324	0,75	0,53093298
1,483		0,81021088
-0,394	0,08	0,19895404
1,11	0,02	0,72874335
0,132	0,17	0,20178026
0,54	0,01	0,37195945
0,07	0,01	0,30243256
-0,48		0,17934586
1,53	0,37	0,77501356
-0,42	0,05	0,17301969
-0,37		0,11426182
0,31	0,03	0,33539846
0,352	0,04	0,63910907
-1,39	0,01	0,03973078
-0,82	0,01	0,0685925
0,591	0,09	0,58131151
-0,011	0,12	0,24528218
-1,18	0,04	0,05560334
1,581	0,52	0,83705176
-0,205	0,69	0,38168506
-0,76		0,11760593
-1,41	0,01	0,03384989
1,12	0,19	0,58454649

-0,237		0,26314611
-1,08	0,05	0,06694299
-1,14		0,04931669
1,12		0,64125774
0,27		0,36572168
0,29	0,04	0,32765359
-0,97	0,02	0,06389548
-0,063		0,19094738
0,337	0,84	0,8048978
1,187	0,02	0,73990782
-0,37		0,15562909
0,674	0,03	0,47232044
1,284	0,02	0,75780382
-0,8	0,05	0,09502646
0,875	0,01	0,44066513
0,22	0,01	0,25449879
-1,84	0,03	0,02336218
-0,24		0,17112357
-0,17	0,01	0,21436987
-0,71	0,23	0,08238651
-1,36	0,01	0,05429971
0,99	0,07	0,5985951
-0,02	0,05	0,23774432
-1,03		0,0654494
-0,82		0,11106409
0,96	0,03	0,43666305
0,579		0,57488329
-0,032	0,03	0,20512394
-0,335		0,23909985
-1,65	0,09	0,04238424
-1,27		0,04721132
0,617	0,24	0,74872879
0,38	0,01	0,36778383
-1,08		0,07704649
0,493		0,28476587
0,007		0,27450747
-0,663	0,13	
-0,57	0,02	0,08344628
-0,5		0,13404178
0,782		0,47595554
0,774	0,03	0,38192943
-1,76		0,03713741
-1,58	0,04	0,03263212
-0,26	0,09	0,10777507
-0,68	0,02	0,19322748
0,51		0,51104047
1,341		0,7515026
1,19	0,06	0,60315695
-0,07	0,04	0,16271828

DN: ERSPC-19BM	DN: PSAD - 19BM	DN: all variables
0,09498918	0,10650962	0,08714667
0,02660687	0,0451745	0,02934311
0,07913573	0,11107706	0,14910415
0,905528	0,6627478	0,889614
0,23135193	0,38375773	0,28056255
0,15323828	0,23472234	0,27645734
0,71835239		
0,09413903		
0,15962976	0,27015837	0,13216776
0,26706186	0,56974698	0,42991653
0,65613634	0,72751195	0,8408114
0,20215251	0,22374698	0,21887603
0,2343566	0,27014986	0,12753702
0,492376	0,55585355	0,40671172
	0,16403974	
0,56508012	0,79664803	0,80965423
	0,49697738	
0,17711996	0,18248354	0,09253026
0,23837146	0,27777377	0,30816494
0,1096683	0,17639371	0,11204435
0,91417289	0,63279872	0,88558889
0,06480927		
0,2150254	0,21814657	0,13222519
0,36142578	0,29798623	0,38405226
0,29927114	0,41651143	0,40152837
0,18710604	0,25254586	0,25592206
	0,1555086	
0,8137894	0,83642672	0,82866614
0,23242807	0,262526	0,34561344
0,04377912	0,37675119	0,21524665
0,78410537	0,80552797	0,74226022
0,30416367	0,40530745	0,43831075
0,64347906	0,65766577	0,68761878
0,40560023	0,37568173	0,28989541
	0,65347355	
0,45975853	0,44328974	
0,03949095	0,09662364	0,10309174
0,17860069	0,24720293	0,25863118
0,02169592	0,06272369	0,01744685
0,38424931	0,32985984	0,34606044
0,05995323	0,13669741	0,05172765
0,05655312	0,07845269	0,03782849
0,78863779	0,88422999	0,77038242
0,14504147	0,2151131	0,08503959
0,07854739	0,10234281	0,04711168
0,03206009	0,04625513	0,02790445

0,0541488	0,06604106	0,05238812
0,06474792	0,08056862	0,04276905
	0,0598269	
0,75503563	0,77577856	0,81146208
0,05000066	0,07135756	0,01754275
	0,15080914	
	0,7625256	
0,08104639	0,1200328	0,12842032
0,42396055	0,43924346	0,5293051
0,05318172	0,08204334	0,05447611
0,96605118	0,82410494	0,97139322
0,67247185	0,57044258	0,47188146
	0,69346686	
	0,82376792	
0,03691628	0,0515484	0,02966147
0,54279248	0,78919489	0,70141047
	0,14259968	
0,03589993	0,07345621	0,04849028
0,03460478	0,0593832	0,01958203
0,18234486	0,06808092	0,17655904
0,87601642	0,84366337	0,90313406
0,16085614	0,13369109	0,06753928
	0,32391771	
0,27668241	0,28630452	0,24825727
0,19945317	0,1424669	0,16260843
	0,38976768	
0,28803218	0,31857984	0,31152919
0,81898474	0,44217803	0,91458941
0,13154038	0,13438822	0,14076076
0,55615756	0,63508899	0,68389243
0,35336198	0,2515751	0,18284222
0,32393814	0,37269076	0,26907336
0,1842269	0,21385105	0,18989375
	0,11753577	
0,93174208	0,71612144	0,89912702
0,11144123	0,12263486	0,11940346
0,26780414	0,26071619	0,24839409
0,29120408	0,49713495	0,45907495
0,0213588	0,03582842	0,01707033
0,05154324	0,08394826	0,03867952
0,43490045	0,53696421	0,53141622
0,2538271	0,42255266	0,22342166
0,03417419	0,04494333	0,02899355
0,96865348	0,7515347	0,95333698
0,80309912	0,26761263	0,70555298
0,02069986	0,04071393	0,01824981
0,74555761	0,58138391	0,66913304

0,041789	0,07932147	0,04367395
	0,05571621	
	0,57253706	
	0,31144032	
0,27115421	0,3827272	0,31255076
0,04296465	0,06090339	0,03413471
	0,21766285	
0,95296407	0,53531523	0,94659136
0,58633157		
	0,13620138	
0,39574905	0,43246889	0,44014817
0,62341518	0,68327849	0,70537042
0,0639065	0,13103361	0,07217355
0,45027399		
0,22306513	0,27731964	0,17045655
0,0115795	0,02204423	0,01279553
	0,1953581	
0,13330023	0,1804658	0,16194547
0,16006421	0,11542279	0,08836773
0,02238584	0,04213528	0,02834189
0,56921773	0,50080736	0,52176685
0,19218068	0,201128	0,15937352
	0,06148383	
	0,12607737	
0,50863273	0,5168688	0,31502055
	0,74701654	
0,17462977	0,18921324	0,13151215
	0,18304795	
0,02086471	0,04883351	0,03231049
	0,04449647	
0,62601154	0,62725957	0,82475779
0,27055425	0,2846302	0,28715048
	0,35522773	
	0,22565175	
0,11176838	0,13537398	
0,07847413	0,10645183	0,03943622
	0,14691474	
	0,45663916	
0,4345873	0,4375029	0,25948315
	0,02199163	
0,01831206	0,03835871	0,01922175
0,16466603	0,14507559	0,06510449
0,0666505	0,11490667	0,1265905
	0,39043149	
	0,7219623	
0,63407364	0,59837586	0,52092111
0,17293674	0,22573806	0,09669085
