# Supplementary figures and images for: Validation of diagnostic nomograms based on CE–MS urinary biomarkers to detect clinically significant prostate cancer
Source: World J Urol. 2022 Jul 16;40(9):2195–203. doi: 10.1007/s00345-022-04077-1 (PMC9427869; doi:10.1007/s00345-022-04077-1)

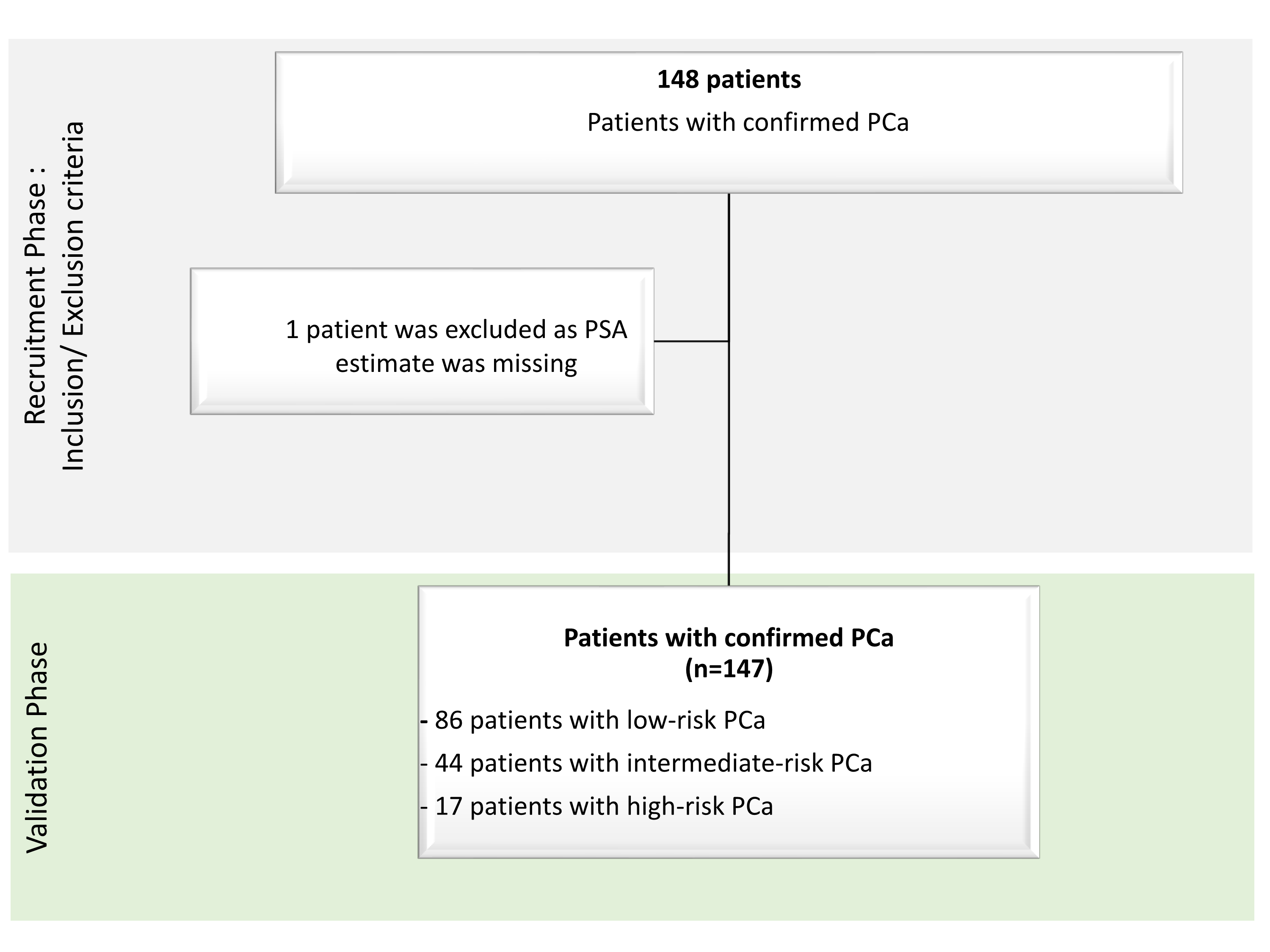

Supplement: Supplementary file 3 — Supplementary file3 Supplementary Figure: Schematic representation of the study design for the validation of urine CE-MS based nomograms. (TIF 230 KB) [file 345_2022_4077_MOESM3_ESM.tif]
