# Supplementary material for: Validation of diagnostic nomograms based on CE–MS urinary biomarkers to detect clinically significant prostate cancer
Source: World J Urol. 2022 Jul 16;40(9):2195–203. doi: 10.1007/s00345-022-04077-1 (PMC9427869; doi:10.1007/s00345-022-04077-1)
Supplement: Supplementary file 4 — Supplementary file4 Supplementary Text: Urine sample processing, mass spectrometry analysis and performance evaluation (DOCX 26 KB) [file 345_2022_4077_MOESM4_ESM.docx]

***Supplementary Text:***

***Urine sample processing and mass spectrometry analysis***

Sample preparation was performed by diluting 700 µl aliquots from the urine collected from PCa patients in two volumes (1:2) alkaline buffer containing 2M urea, 10mM NH_4_OH and 0.02% SDS (pH 10.5). The samples were subsequently filtered by Centrisart ultracentrifugation filters (Sartorius, Göttingen, Germany) to retain proteins/ polypeptides below 20kDa that were further desalted through PD-10 columns (GE Healthcare, Munich, Germany). CE-MS analysis and data processing was performed according to ISO13485 standards yielding quality controlled urinary data sets[^1^](#_ENREF_1). Mass spectral ion peaks representing identical molecules at different charge states were de-convoluted into single masses using MosaiquesVisu software[^1^](#_ENREF_1)^,^[^2^](#_ENREF_2). A peak list of each peptide was defined by its molecular mass (kDa), normalized migration time (min) and normalized signal intensity (AU)[^2^](#_ENREF_2). Normalization of the CE-MS data was based on 29 internal collagen fragments stable over disease/ health state that serve as internal standards[^3^](#_ENREF_3). All detected peptides were deposited, matched, and annotated in a Microsoft SQL database[^4^](#_ENREF_4) and used as input in the presented study. These data have not been previously described before and are unique to this study. Transformation of the data (log-transformation) was performed before performing the statistical analysis, as previously described[^5^](#_ENREF_5).

***Statistical analysis and performance evaluation***

Sensitivity and specificity for the SVM-based peptide marker pattern were calculated based on the number of correctly classified samples, as defined by biopsy, considering the previously reported cut-off criterion of (-0.07). Receiver operating characteristic (ROC) plots and the respective confidence intervals (95%CI) were based on exact binomial calculations and were calculated in MedCalc 12.7.5.0 (Mariakerke, Belgium). The area under the ROC curve (AUC) was evaluated to estimate the overall accuracy independent upon a particular threshold[^6^](#_ENREF_6), and the values were then compared using DeLong tests. Statistical comparisons of the classification scores between the PCa risk groups and GS groups were performed by the Kruskal-Wallis rank sum test using MedCalc 12.7.5.0 (Mariakerke, Belgium)[^7^](#_ENREF_7). The diagnostic nomograms (DN) of 19-BM in combination with clinical variables were established using multiple linear regression analyses. Decision curve analysis (DCA)[^8^](#_ENREF_8) examined the potential net benefit of using the diagnostic nomograms in the clinic, according to which a net benefit is defined as a function of the decision threshold at which one would consider obtaining a biopsy.

**References**

1. Zurbig P, Renfrow MB, Schiffer E, Novak J, Walden M, Wittke S *et al*. Biomarker discovery by CE-MS enables sequence analysis via MS/MS with platform-independent separation. *Electrophoresis* 2006; **27**(11)**:** 2111-2125.

2. Frantzi M, Metzger J, Banks RE, Husi H, Klein J, Dakna M *et al*. Discovery and validation of urinary biomarkers for detection of renal cell carcinoma. *J Proteomics* 2014; **98:** 44-58.

3. Siwy J, Mullen W, Golovko I, Franke J, Zurbig P. Human urinary peptide database for multiple disease biomarker discovery. *Proteomics Clin Appl* 2011; **5**(5-6)**:** 367-374.

4. Latosinska A, Siwy J, Mischak H, Frantzi M. Peptidomics and proteomics based on CE-MS as a robust tool in clinical application: The past, the present, and the future. *Electrophoresis* 2019; **40**(18-19)**:** 2294-2308.

5. Dakna M, Harris K, Kalousis A, Carpentier S, Kolch W, Schanstra JP *et al*. Addressing the challenge of defining valid proteomic biomarkers and classifiers. *BMC Bioinformatics* 2010; **11:** 594.

6. DeLeo JM. Receiver operating characteristic laboratory (ROCLAB): Software for developing decision strategies that account for uncertainty. *Uncertainty Modeling and Analysis, 1993 Proceedings, Second International Symposium on* 1993**:** 318-325.

7. Roobol MJ, van Vugt HA, Loeb S, Zhu X, Bul M, Bangma CH *et al*. Prediction of prostate cancer risk: the role of prostate volume and digital rectal examination in the ERSPC risk calculators. *European urology* 2012; **61**(3)**:** 577-583.

8. Vickers A, Elkin E. Decision Curve Analysis: A Novel Method for Evaluating Prediction Models. . *Medical Decision Making* 2006; **6:** 565-574.
